# An Intelligent Rice Yield Trait Evaluation System Based on Threshed Panicle Compensation

**DOI:** 10.3389/fpls.2022.900408

**Published:** 2022-07-22

**Authors:** Chenglong Huang, Weikun Li, Zhongfu Zhang, Xiangdong Hua, Junya Yang, Junli Ye, Lingfeng Duan, Xiuying Liang, Wanneng Yang

**Affiliations:** National Key Laboratory of Crop Genetic Improvement, National Center of Plant Gene Research (Wuhan), College of Engineering, Huazhong Agricultural University, Wuhan, China

**Keywords:** rice panicle, yield traits, high-throughput, Faster-RCNN, cloud computation

## Abstract

High-throughput phenotyping of yield-related traits is meaningful and necessary for rice breeding and genetic study. The conventional method for rice yield-related trait evaluation faces the problems of rice threshing difficulties, measurement process complexity, and low efficiency. To solve these problems, a novel intelligent system, which includes an integrated threshing unit, grain conveyor-imaging units, threshed panicle conveyor-imaging unit, and specialized image analysis software has been proposed to achieve rice yield trait evaluation with high throughput and high accuracy. To improve the threshed panicle detection accuracy, the Region of Interest Align, Convolution Batch normalization activation with Leaky Relu module, Squeeze-and-Excitation unit, and optimal anchor size have been adopted to optimize the Faster-RCNN architecture, termed ‘TPanicle-RCNN,’ and the new model achieved F1 score 0.929 with an increase of 0.044, which was robust to indica and japonica varieties. Additionally, AI cloud computing was adopted, which dramatically reduced the system cost and improved flexibility. To evaluate the system accuracy and efficiency, 504 panicle samples were tested, and the total spikelet measurement error decreased from 11.44 to 2.99% with threshed panicle compensation. The average measuring efficiency was approximately 40 s per sample, which was approximately twenty times more efficient than manual measurement. In this study, an automatic and intelligent system for rice yield-related trait evaluation was developed, which would provide an efficient and reliable tool for rice breeding and genetic research.

## Introduction

Rice is the staple food for over half of the world’s population (Zhang, 2007; Sandhu et al., 2019), the yield of which is of great significance to human security and development (Tester and Langridge, 2010). In rice research, the evaluation of rice yield-related traits is an essential step for rice breeding (Qian et al., 2016; Yang et al., 2020) and functional gene analysis (Sakamoto and Matsuoka, 2008). Generally, rice yield is determined by crucial factors, including the number of panicles (Xing and Zhang, 2010), the number of filled spikelet’s (Borrell et al., 2014), and 1,000 grain weight (Richards, 2000). However, the traditional method for rice yield-related trait evaluation is manual and faces the problems of rice threshing difficulties, measurement process complexity, and low efficiency. Therefore, an efficient and reliable tool for rice yield-related trait evaluation is urgently needed.

With the rapid development of machine vision, a growing number of image-based technologies have been applied in agriculture (Sankaran et al., 2010; Rebolledo et al., 2016; Confalonieri et al., 2017), and several studies on rice yield-related trait evaluation have been reported. Since it is difficult to thresh all the spikelet’s in the panicle, most of the studies focus on grain traits measurement. ImageJ, a Java-based open source software, was used for isolated grain trait measurement (Igathinathane et al., 2009), and Smart Grain, an open source software, was released for grain trait measurement in complicated situations (Tanabata et al., 2012). Duan developed a yield traits scorer for automatic extraction of yield-related traits with high throughput (Duan et al., 2011). However, panicle threshing performance is still a bottleneck that some unfilled spikelet’s would remain in the threshed panicle, which would have a great negative effect on the measurement of rice yield-related traits. Some researchers have attempted to directly analyze rice panicles without threshing processes. Sandhu proposed a method for rice panicle maturity evaluation based on three-dimensional point cloud construction and analysis (Sandhu et al., 2019). Hu developed a 22 yield-related trait extraction method based on X-ray computed tomography imaging (Hu et al., 2020). P-TRAP, a commercial software program, was designed for yield spread-related trait analysis (Al-Tam et al., 2013). However, the low measurement efficiency and high cost had a negative impact on the practical application. Thus, an automatic panicle analysis system with high efficiency and high accuracy would have great application prospects.

According to existing research, there are generally two ways to obtain rice yield traits. The most common method was to investigate the grain traits by threshing the panicle manually (Yang et al., 2014), because the unfilled spikelet’s were hard to be completely taken off by the threshing machine, which limited the efficiency and accuracy of rice yield traits extraction. On the other hand, the X-ray technology was able to be used for filled and unfilled spikelet’s identification (Hu et al., 2020), but it is difficult to be widely used in practical rice yield traits extraction, because of the high cost, low efficiency, and radiation. In order to solve the problem of residual panicle spikelet’s by threshing machine, we promote a new way with threshed panicle compensation, which would identify the number of residual spikelet’s in the threshed panicle and compensate it into the evaluation of spikelet yield traits. In order to achieve it, a robust and reliable method for threshed panicle identification is needed.

In recent years, artificial intelligence technology has been significantly promoted and widely used in agriculture (Chen et al., 2018; Escamilla-Garcia et al., 2020; van Klompenburg et al., 2020; Dhaka et al., 2021; Kattenborn et al., 2021). Sun developed a soybean yield prediction model based on a convolutional neural network (Sun et al., 2019) and a long short-term memory network (Hochreiter and Schmidhuber, 1997). Zhou analyzed drone images for maize leaf coverage based on the Deeplabv3 plus model (Zhou et al., 2019). Object detection is an important research field of deep learning image processing, which is widely used in various agricultural scenes. The state-of-the-art methods can be categorized into two main types: one-stage methods and two stage-methods. One-stage methods prioritize inference speed, and example models include YOLO, SSD, and Retina (Liu et al., 2016; Redmon et al., 2016; Lin et al., 2020). Two-stage methods prioritize detection accuracy, and example models include Faster R-CNN, Mask R-CNN, and Cascade R-CNN (Ren et al., 2015; He et al., 2017; Cai et al., 2018). Faster RCNN is a classical two-stage object detector consisting of object proposal, feature extraction, and bounding box regression, which formulates detection as a coarse-to-fine process. The Deep–Fruits model was proposed for fruit identification based on Faster-RCNN (Sa et al., 2016). Faster-RCNN has also been applied for pest detection (Shen et al., 2018; He et al., 2020; Li et al., 2021) and panicle spikelet counting (Wu et al., 2019; Deng et al., 2021; Yu et al., 2021). Therefore, we adopted a two-stage detector, Faster R-CNN, as the basis for the threshed panicle detection. However, the current Faster R-CNN detector has shown weak performances with small and overlapped objects (Tong et al., 2020), and the grain size is approximately 30 × 60 pixels in the threshed panicle image of 2,048 × 4,096 pixels. Building on these preliminary observations, appropriate improvement for the Faster R-CNN architecture should be performed to achieve accurate detection of the threshed panicle.

The aim of this study is to build an automatic and intelligent system for rice yield trait evaluation. Firstly, a new deep learning architecture was proposed to achieve threshed panicle compensation on the basis of the Faster R-CNN architecture, termed ‘TPanicle-RCNN.’ Then, equipped with automatic control, machine vision, and deep learning algorithms, we developed a novel intelligent system, which includes an integrated threshing unit, grain conveyor-imaging units, and threshed panicle conveyor-imaging unit, and specialized image analysis software. Finally, the threshed panicle compensation was performed to achieve automatic and accurate acquisition of rice yield-related traits.

## Materials and Methods

### System Design

**FIGURE 1:**
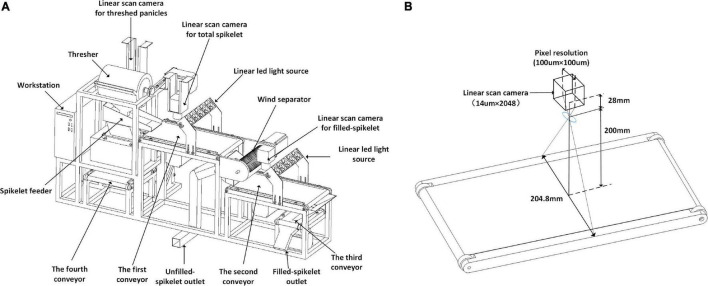
System sketch of automatic rice yield-related trait evaluation: **(A)** system composition details and **(B)** conveyor imaging unit.

The system sketch is shown in Figure 1A, which mainly consists of the panicle threshing unit, servo air separator, and three conveyor imaging units. The threshing unit is derived from a semi-feeding drum thresher (TSL-150A, Top Cloud-AGRI, China) and is driven by a servo motor (Panasonic, Japan). The operator holds the panicle and puts it into the thresher; as a result, all the filled spikelet’s and most of the unfilled spikelet’s are threshed from the panicle. Then, the threshed spikelet’s spread out on the first conveyor by the vibration feeder. The servo air separator is designed with a cross-flow fan and is fixed between two grain conveyor lines; as a result, the filled spikelet’s are separated from the unfilled spikelet’s. The conveyor-imaging units are constructed with a panicle conveyor line and two grain conveyor lines; as a result, images of threshed panicles, total spikelet’s, and filled spikelet’s are obtained, which were grayscale images saved in PNG format. Finally, the filled spikelet’s and unfilled spikelet’s are individually collected from the specific outlets.

The details of the conveyor imaging units are shown in Figure 1B. The linear camera (SG-11-02K40-00-R, DASLA, Canada) is equipped with a 28 mm lens (Nikon, Japan), the charge-coupled device (CCD) size is 14μ*m*×2048, and the field of vision (FOV) is 204.8 mm with a pixel resolution of 100μ*m*. Motion control is conducted by a programmable logic controller (PLC, Omron, Japan) programed by CX-Programmer 9.5 (Omron, Japan). The workstation m415 (Lenovo, China) is equipped with an i5-7500 CPU, 8 GB memory, and 1 T hard disk, and the Alibaba cloud (Alibabacloud) is adopted, with the configuration of a Tesla M40, 16 GB memory, and 30 GB cloud storage.

### System Workflow

**FIGURE 2:**
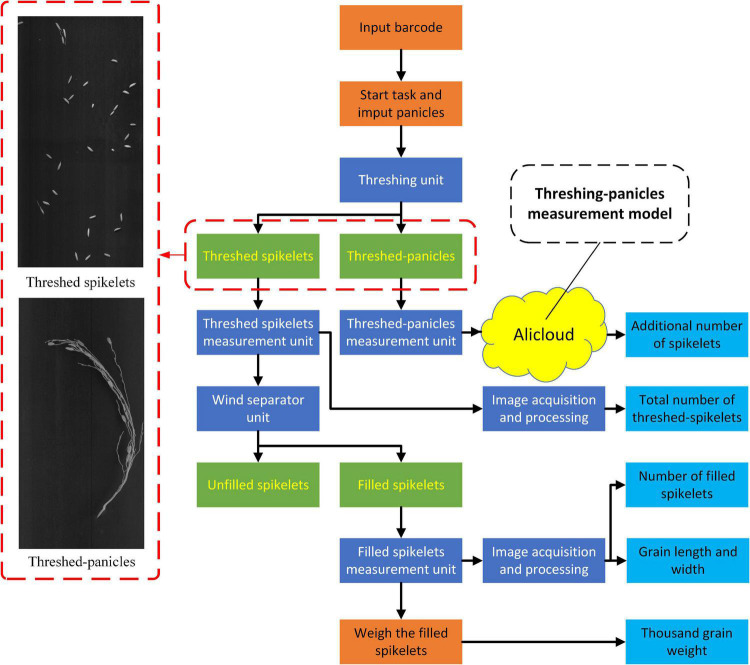
System workflow for yield related trait measurement.

The system workflow is depicted in Figure 2: (1) First, the operator started the system and inputs the barcode. (2) Second, the panicle is held and put into the thresher after which the threshed panicle is placed on the fourth conveyor line for image acquisition, while all the threshed grains spread out onto the first conveyor line with a spikelet’s feeder for image acquisition. (3) Then, the grains go through a wind separator that blows the unfilled spikelet’s away, while the filled spikelet’s falls onto the second conveyor for image acquisition. (4) Next, all the filled spikelet’s are collected and weighed by the third conveyor and weighing device. (5) Finally, the images of threshed panicles, total spikelet’s, and filled spikelet’s are analyzed by specific local algorithms and cloud-deployed deep learning models for yield-related trait evaluation. The system workflow is shown in Supplementary Video 1.

### Software Design

**FIGURE 3:**
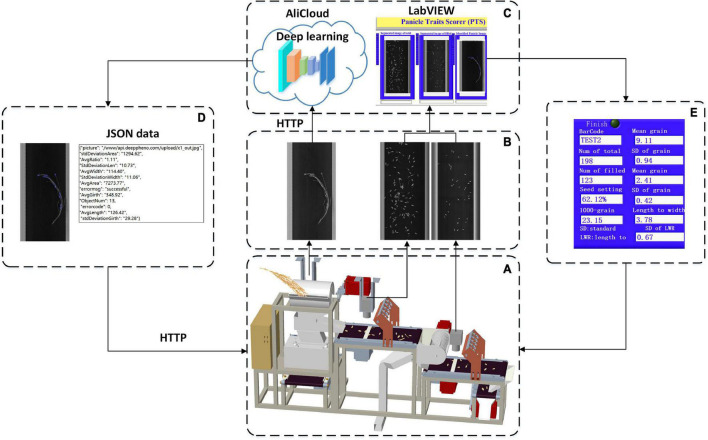
Software workflow in the system: **(A)** system control, **(B)** image acquisition, **(C)** image analysis, **(D)** cloud communication, and **(E)** result exhibition.

The software workflow in the system is shown in Figure 3 and was developed to achieve the functions of PLC communication, image acquisition, image analysis, and data storage. First, PLC communication was designed to control the panicle thresher, vibration feeder, conveyor lines, and wind separator, as shown in Figure 3A. Second, image acquisition was achieved by the NI-Vision module (National Instruments, United States) to obtain images of threshed panicles, total spikelet’s, and filled spikelet’s, as shown in Figure 3B. The image analysis algorithms were written in C + + and complied with the Dynamic Link Library (DLL), which was invoked by the user software developed by LabVIEW 8.6 (National Instruments, United States), and the threshed panicle identification model was trained in the local server and deployed in the Alibaba cloud. Finally, as shown in Figure 3E, the measurement results were displayed on the software interface, and the data were saved in one Excel file.

#### Pipeline for the Grain Image Analysis

**FIGURE 4:**
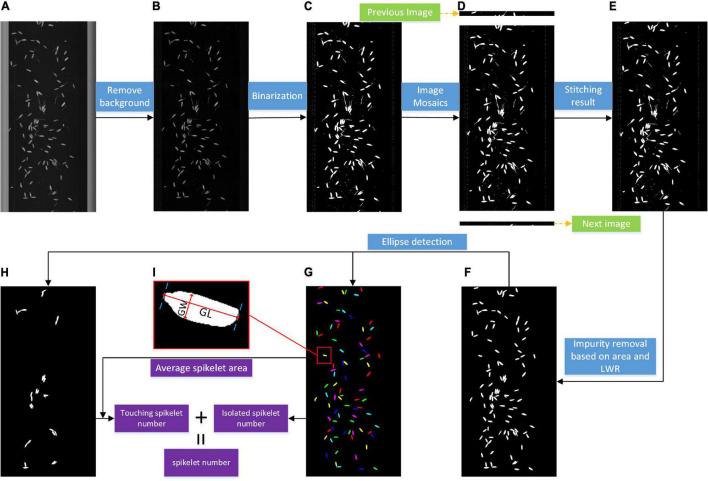
Pipeline for the grain image analysis.

The grain image analysis pipeline is presented in Figure 4. First, background subtraction was conducted to enhance the foreground contrast shown in Figure 4B. Second, a fixed threshold was applied to obtain the binary image shown in Figure 4C. Because of continuous linear scanning, the grain was probably distributed on two adjacent images; therefore, sequence image stitching was implemented based on the bottom connected region shown in Figure 4D, and a binary image with complete grains (Figure 4E) was obtained. Then, the impurity removal algorithm was carried out based on the optimal thresholds of the area and length width ratio (LWR), as shown in Figure 4F, which was determined by the distribution of the grain size and a large number of experiments. Next, ellipse detection was used to obtain the isolated grain image (Figure 4G) and touching grain image (Figure 4H), and the range of minor and major axis lengths was set according to grain size; the minimum match score was set to 800, while 1,000 represented a perfect match.

Based on the above image processing, the number of connected regions in the isolated grain image was taken as the isolated spikelet number, while the areas of the connected regions in the touching grain image were divided by the average area of isolated grains, and the sum of the results was regarded as the touching spikelet number. The total number was calculated by adding the isolated spikelet number and touching the spikelet number. The filled spikelet number could also be calculated. Moreover, the grain shape parameters were extracted by the following contour computation method based on the filled isolated grain image. The Euclidean distance between the two farthest points of the contour is regarded as the grain length (GL), and the maximum perpendicular distance is regarded as the grain width (GW).

#### Pipeline for the Threshed Panicle Analysis

Since Faster-RCNN is a classical two-stage object detector that formulates detection as a coarse-to-fine process, the deep learning architecture for threshed panicle analysis was proposed on the basis of Faster-RCNN, termed ‘TPanicle-RCNN,’ while two feature extraction networks, VGG (Simonyan and Zisserman, 2014) and RESNET (He et al., 2016), were studied. To construct the model, 1,072 rice panicles, including 536 indica and 536 japonica panicles, were threshed by the threshing unit, a total of 1,072 threshed panicle images were captured and manually labeled with Labelimg (LabelImg.), and all the ground-truth bounding boxes and annotation files saved in the PASCAL VOC data format (Krizhevsky et al., 2012). Then the dataset was divided into a training set and a test set at a ratio of 4:1, with half indica and half japonica in each data set. Then, the datasets were augmented four times by image flipping and brightness adjustment, while 3,432 training images and 856 testing images were obtained. All the training and testing data are available at https://pan.baidu.com/s/1-XawHGseIc5bboVOP48Fkw?pwd=153w with the extraction code ‘153w’ for non-commercial research purposes.

#### Model Improvement

**FIGURE 5:**
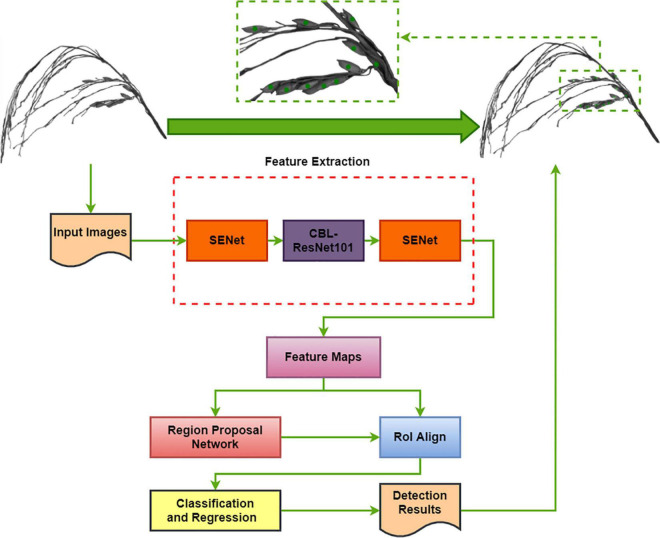
Block diagram of the TPanicle-RCNN model. The dotted red rectangles indicate the model improvements.

The Block diagram of the TPanicle-RCNN model is depicted in Figure 5, while the red rectangles indicate the model improvements compared with the original Faster R-CNN architecture. The RoIPool operation had adopted two quantization processes, which would result in a deviation of the ultimate box, while the location error would put a great negative effect on the threshed panicle detection, especially when the grains were close or slightly overlapped. To improve it, the Region of Interest Align (RoIAlign) designed in Mask R-CNN architecture, was applied to calculate the exact values of the candidate-box coordinates in this method. The original Faster R-CNN architecture used nine anchors to detect regional proposals consisting of three scales 128^2^, 256^2^, and 512^2^, and three aspect ratios, 1:1, 1:2, and 2:1, which were not suitable for the small object detection. Therefore the scale vectors were optimized to 32^2^, 64^2^, and 128^2^ based on the sizes of the grain. The integration of Convolution, Batch normalization, and Leaky Relu (CBL) was used to replace the traditional convolution and activation in the Res101 network (Wu and He, 2018), which was helpful to speed up the training efficiency and improve the accuracy. Moreover, the squeeze-and-excitation unit had been embedded into the head and tail of the feature extraction net Res101^[48]^, and the channel attention layer had merged global average pooling and maximum pooling, which was able to enhance the effective information and improve model accuracy. In conclusion, the RoIAlign, CBL module, Squeeze-and-Excitation unit, and optimal anchor size had been adopted in the TPanicle-RCNN model.

#### Model Training

Pytorch 1.7.1 was adopted as a deep learning framework, and python 3.6 was applied. Since the training dataset was small, the fine-tuning training technique had been applied. The Faster-RCNN and TPanicle-RCNN models were initialized with pre-trained weights obtained by training the ImageNet dataset (Ioffe and Szegedy, 2015). And then the models were trained on the 3,432 training set to further optimize the pre-trained net. During the fine-tuning stage, the batch-size was set to four to achieve the maximum utilization of graphic processing unit GPU memory. The intersection-over-union (IoU) was set to 0.5, which means that when its IoU was ≥ 0.5, the predicted bounding box was regarded as positive. The number of region proposals was set to 600, the shortest side of the image was set to 1,000 pixels, and the longest side of the image was set to 2,000 pixels, in accordance with grain number and image size. Other parameters were configured by the default settings of the Faster R-CNN network. Finally, the optimal model was deployed on Alibaba cloud for threshed panicle analysis, and the spikelet number was returned as the total spikelet compensation.

### System Evaluation

To evaluate the threshed panicle model performance, 856 testing images were tested, and the indicators of precision, recall, PR curve, *F*1 values, and average precision (AP) were used, the calculations of which are shown in Equations 1–4. *T*_*P*_ represents the true positive targets, where the targets were correctly identified, *F*_*P*_ represents the false positive targets, where the background was identified as the target, and *F*_*N*_ represents the false negative targets, where the targets were missed.


(1) $$P\,r\,e\,c\,i\,s\,i\,o\,n=\frac{T_{P}}{T_{P}+F_{P}}$$

(2) $$R\,e\,c\,a\,l\,l=\frac{T_{P}}{T_{P}+F_{\text{N}}}$$

(3) $$F\,1=\frac{2\times P\,r\,e\,c\,i\,s\,i\,o\,n\times R\,e\,c\,a\,l\,l}{P\,r\,e\,c\,i\,s\,i\,o\,n+R\,e\,c\,a\,l\,l}$$

(4) $$A\,P=\int_{0}^{1}P\,\left(r\right)\,dr$$

To evaluate the system accuracy, 504 randomly selected panicle samples were tested, and the results of the system measurement were compared with manual measurements, including the threshed panicle spikelet number (TPSN), unfilled spikelet number, and filled spikelet number, and the mean value of three manual measurements was taken as the ground truth. Additionally, the indicators of R square, the mean absolute percentage error (MAPE), and the root mean square error (RMSE) were computed using Equations 5–7 to evaluate the system performance. Additionally, 200 randomly selected panicle samples were tested to evaluate the system efficiency.


(5) $$R^{2}=\frac{\sum_{i=1}^{n}{\left(X_{a\,i}-\bar{X_{m}}\right)}^{2}}{\sum_{i=1}^{n}{\left(X_{m\,i}-\bar{X_{m}}\right)}^{2}}$$

(6) $$M\,A\,P\,E=\frac{1}{\text{n}}\,\sum_{i=1}^{n}\frac{\left|X_{a\,i}-X_{m\,i}\right|}{X_{m\,i}}$$

(7) $$R\,M\,S\,E=\sqrt{\frac{1}{\text{n}}\,\sum_{i=1}^{n}{\left(X_{a\,i}-X_{m\,i}\right)}^{2}}$$

where n is the total number of measurements; *X*_*mi*_ is the manual measurement results; *X*_*ai*_ is the system measurement results; and $\bar{X_{m}}$ is the mean of the manual measurements.

## Results

**FIGURE 6:**
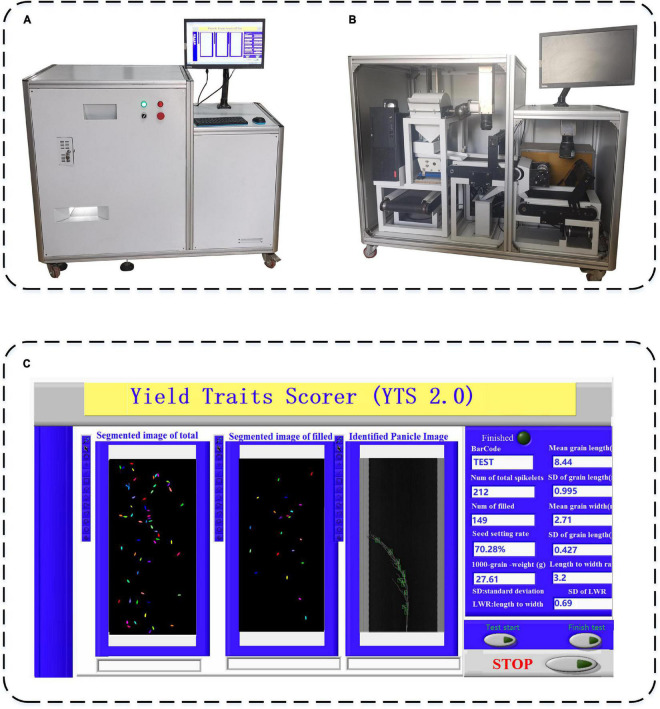
The prototype of an automatic rice yield-related trait evaluation system, **(A)** system hardware composition, **(B)** system internal structure, and **(C)** system software interface.

The prototype of the automatic rice yield-related trait evaluation system is shown in Figure 6. The system hardware composition is shown in Figure 6A, and the details of the system’s internal structure are depicted in Figure 6B, while the system software interface is exhibited in Figure 6C. From the results, the specific functions of system hardware and software were realized, and images of threshed panicles, total spikelet’s, and filled spikelet’s were analyzed for rice yield-related traits, including the total spikelet number, seed setting rate, grain shape, and grain weight. To demonstrate the system, the performance with different object detection models and rice varieties were studied, and furthermore, the accuracy and efficiency of the system were evaluated in detail.

### Evaluation of Models on the Testing Dataset

**FIGURE 7:**
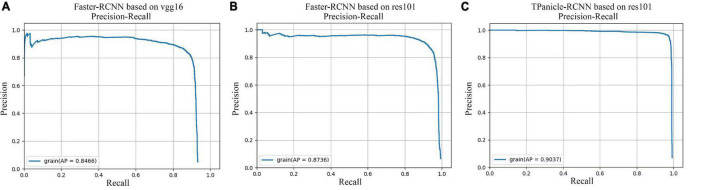
Comparisons of precision–recall curves, **(A)** Faster-RCNN based on vgg16, **(B)** Faster-RCNN based on ResNet101, **(C)** TPanicle-RCNN based on ResNet101.

The models were evaluated by the 856 testing dataset, while comparisons of precision–recall curves of Faster R-CNN and TPanicle-RCNN models are shown in Figure 7, which showed that the average precision (AP) with the IoU ≥ 0.5 was 0.836, 0.873, 0.903 for the Faster-RCNN based on vgg16 (Figure 7A), resl01 (Figure 7B), and TPanicle-RCNN based on ResNet101 (Figure 7C), respectively. The results indicated that the res101 network had better performance than vgg16 in the feature extraction of small objects, therefore the Faster-RCNN improvement was based on ResNet101. Based on the advantages of RoIAlign, CBL module, Squeeze-and-Excitation unit, and optimal anchor size, the precision of TPanicle-RCNN was significantly greater than that of the original Faster R-CNN under the same recall conditions for threshed panicle, and the AP has reached 0.903, with an increase of 0.03. With the confidence threshold set as 0.5, the *F*1 value of improved Faster-RCNN was 0.929, an increase of 0.044.

### The Performance for Indica and Japonica

The threshed panicle identification performance for the indica and japonica varieties in the testing dataset was analyzed to evaluate the reliability and flexibility of the system, and the results are shown in Table 1 with 428 indica and 428 japonica samples. With the Faster-RCNN based on ResNet101, the recall, precision, and F1 scores were 0.832, 0.856, and 0.844 for indica varieties, while the recall, precision, and F1 scores were 0.914, 0.936, and 0.925 for japonica varieties, respectively. With the TPanicle-RCNN, the recall, precision, and F1 scores were 0.881, 0.900, and 0.891 for indica varieties, while the recall, precision, and F1 scores were 0.975, 0.958, and 0.967 for indica varieties, respectively. From the results, the TPanicle-RCNN yielded better performance than the original Faster-RCNN in general, the improvements in recall, and precision was 0.049, 0.044 for indica, and 0.061, 0.018 for japonica, which indicated that the TPanicle-RCNN was able to greatly improve the recall rate and recognition accuracy of the threshed panicle. Additionally, the models performed better on the japonica varieties than the indica varieties, because the spikelet number in the japonica threshed panicles was obviously less than that in the indica threshed panicles, which led to an increase in the recall rate, and in the results, the average gap between the *F*1 values was 0.076 for the improved Faster-RCNN.

**TABLE 1:** The performance of the threshed panicle identification models.

Class	Data	Model	Recall	Precision	*F*1
Indica	428	Faster-RCNN	0.832	0.856	0.844
		TPanicle-RCNN	0.881	0.900	0.890
Japonica	428	Faster-RCNN	0.914	0.936	0.925
		TPanicle-RCNN	0.975	0.958	0.967

**FIGURE 8:**
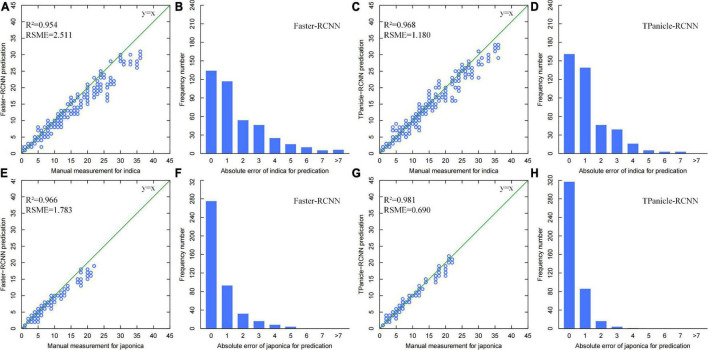
**(A,B)** Threshed panicle spikelet number scatter plots of manual versus model measurement and error distribution diagrams for indica rice samples with Faster-RCNN measurement (Brichet et al., 2017), TPanicle-RCNN measurement **(C,D)**, and japonica rice samples with Faster-RCNN measurement **(E,F)**, TPanicle-RCNN measurement **(G,H)**.

The threshed panicle spikelet number (TPSN) was measured manually and identified by the model inference, while the scatter plots of manual *vs.* model measurement and error distribution diagrams for the 428 indica and 428 japonica rice samples are shown in Figure 8, without positive and negative sample division based on IoU. From the results, the R^2^ and RMSE of the improved Faster-RCNN were 0.968 and 1.18, respectively, for the indica varieties, with an improvement of 0.014 and 53%. Meanwhile, the R^2^ and RMSE of the improved Faster-RCNN were 0.981 and 0.690, respectively, for the japonica varieties, with an improvement of 0.015 and 61.23%. The results demonstrated high consistency with manual measurements and a significant improvement compared with the original Faster-RCNN. The error distribution diagrams also indicated that the error distribution was normal in general, and 80% of identification errors constituted fewer than 2 spikelet’s. On the basis of these results, the error distribution of indica varieties was more discrete than that of japonica, and the improved Faster-RCNN has greatly decreased the error, which was preferred and used in the system.

### Accuracy of the Automatic Rice Yield-Related Trait Evaluation System

**FIGURE 9:**
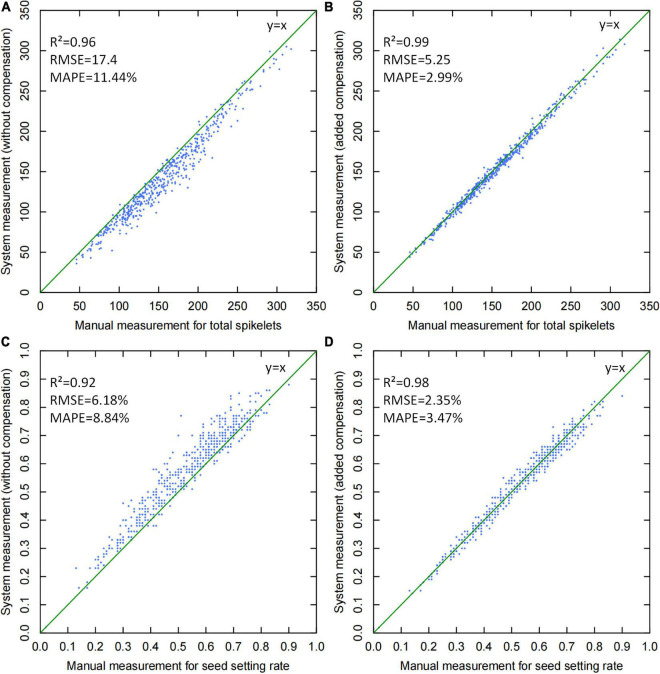
Scatter plots of manual versus system measurements for yield-related trait evaluation: **(A)** total spikelet measurement without threshed panicle compensation, **(B)** total spikelet measurement with threshed panicle compensation, **(C)** seed setting rate measurement without threshed panicle compensation, and **(D)** seed setting rate measurement with threshed panicle compensation.

To test the system accuracy, 504 randomly selected panicle samples were measured, and the scatter plots of manual *vs.* system measurements are shown in Figure 9. From the results, yield-related traits, including total spikelet number and seed setting rate, were mainly analyzed to evaluate the effect of threshed panicle compensation. If threshed panicle compensation was not conducted, the R^2^ and MAPE of the total spikelet measurement were 0.96 and 11.44%, respectively, which were improved to 0.99 and 2.99% by threshed panicle compensation. Additionally, the R^2^ and MAPE of the seed setting rate measurement were 0.92 and 8.84%, respectively, which were improved to 0.98 and 3.47% by threshed panicle compensation. In conclusion, the accuracy of the entire system was significantly improved by threshed panicle compensation, enhancing the reliability of the automatic rice yield-related trait evaluation system.

### System Efficiency Evaluation

**FIGURE 10:**
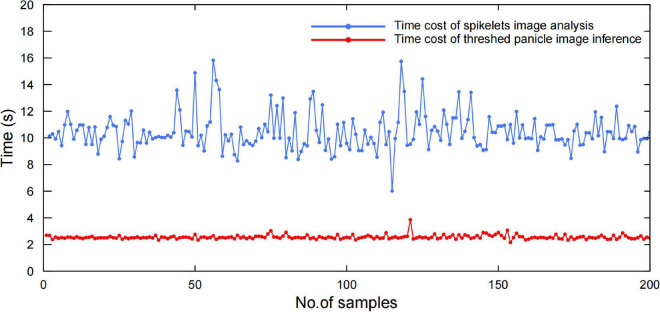
Efficiency evaluation with 200 randomly selected panicle samples.

To evaluate the system efficiency, 200 randomly selected panicle samples were tested, while the time costs of panicle threshing, image acquisition, and image analysis were recorded individually. The results proved that the average efficiency was approximately 40 s, in which the time costs of panicle threshing, panicle image acquisition, and image analysis were approximately 25, 5, and 10 s, respectively, as shown in Figure 10. Additionally, the spikelet image analysis was performed in parallel with the panicle analysis, in which the time cost was approximately 4 s. Therefore, the whole system efficiency was approximately 40 sper panicle, which was approximately 20 times more efficient than manual measurement.

## Discussion

**FIGURE 11:**
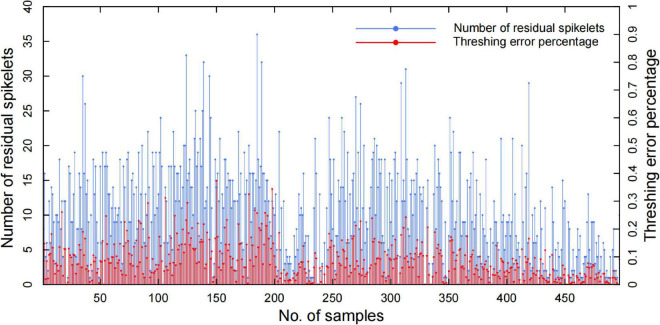
Automatic threshing performance of the residual spikelet number and threshing error percentage.

The results demonstrated that the system measurement accuracy mainly depended on the threshed panicle compensation, which had a high correlation with the threshing effect. To evaluate the threshing effect, the spikelet’s in the threshed panicle, unfilled spikelet outlet, and filled spikelet outlet were counted manually to obtain the threshing error percentage, and the results of 504 panicle samples are shown in Figure 11. The threshing results demonstrated that the filled spikelet’s were able to be threshed well but a few unfilled spikelet’s remained in the panicle, and the average spikelet number in the threshed panicle was 10.48, which led to a 9.37% average threshing error percentage. Additionally, the results showed great fluctuation in the threshing performance due to the panicle type and threshing time. In general, the threshing error contributed 81.9% of the total spikelet measurement error, while the threshed panicle compensation decreased the threshing error by 90.18%, which was of great significance to automatic yield-related trait evaluation.

**FIGURE 12:**
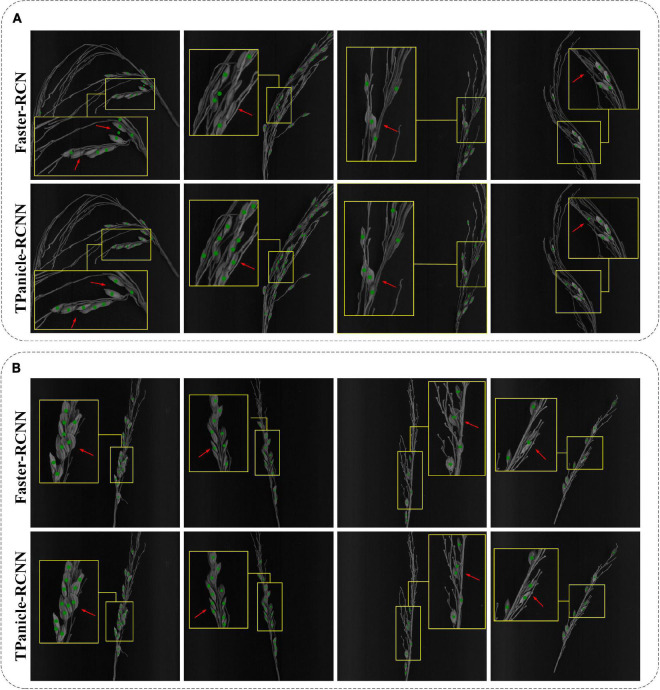
Threshed panicle identification of the indica **(A)** and japonica **(B)** subspecies for the Faster-RCNN model and TPanicle-RCNN model. The red arrow indicates the comparison of the identification results by different models.

Compared with the original Faster-RCNN architecture, it was demonstrated that the TPanicle-RCNN had significantly increased the performance, and the detailed prediction cases are shown in Figure 12, including indica varieties (Figure 12A) and japonica varieties (Figure 12B). The results proved that the CBL module, Squeeze-and-Excitation unit, and optimal anchor size were able to help extract more effective features, so as to improve the model accuracy, while the RoIAlign was able to significantly improve the regression accuracy of the target box. Therefore the TPanicle-RCNN had performed a higher recall rate and grain position accuracy, which was indicated by the red arrow in Figure 12. Regarding the performance of the japonica and indica varieties, the results showed that the spikelet’s in indica threshed panicles were dense, and the threshed panicle structure was more complex, while the japonica panicles were easier to thresh. Therefore, the recall rate and precision value of indica varieties were lower than that of japonica varieties. Overall, the TPanicle-RCNN had a better performance of adaptability and reliability, regardless of panicle varieties and density.

According to the time cost of each step, the system efficiency was mainly decided by the threshing time, and the share of threshed panicle image inference was approximately one in eight, which indicated that the cloud computing mode did not distinctly decrease the system efficiency. In contrast, cloud computing dramatically reduced the system cost and improved the system flexibility. Thus, this study demonstrated a novel automatic system for rice yield-related trait evaluation with high accuracy and efficiency, which was of great significance to rice breeding and genetic research.

In the past studies on rice yield traits evaluation, it was difficult to balance the accuracy, automation, and practicality. For example, using X-ray computed tomography to analyze rice panicle traits could reach an R2 of 0.98 for grain number (Hu et al., 2020). However, this method required 2 min to scan and reconstruct each panicle, while the cost and radiation risk limited the practical application. We have also tried to design threshing equipment that could improve the threshing performance, and the threshing error is about 5%. However, the complicated mechanical structure and threshing error limited its popularization (Huang et al., 2013). Therefore, the existing research and equipment are still unable to meet the needs of the practical rice yield traits evaluation with high accuracy and efficiency. In this research, we have demonstrated an intelligent method that could solve the threshing problem by threshed panicle compensation, and provide an efficient and reliable tool for rice breeding and genetic research.

## Conclusion

This study developed a novel automatic system, for rice yield-related trait evaluation based on the technologies of automatic control, machine vision, and deep learning, in which the threshing problem has been skillfully solved by threshed panicle compensation. Moreover, a new deep learning architecture for threshed panicle analysis was proposed on the basis of Faster-RCNN, termed ‘TPanicle-RCNN’ and deployed in the cloud, which increased automation and improved measurement accuracy. The TPanicle-RCNN was improved by integration of the RoIAlign, CBL module, Squeeze-and-Excitation unit, and optimal anchor size, while various datasets were used to evaluate the threshed panicle identification model. The results indicated that the TPanicle-RCNN showed good performance on both japonica and indica varieties, while the F1 score was 0.929 with an increase of 0.044. To evaluate the system accuracy, 504 panicle samples were tested, and the total spikelet measurement error decreased from 11.44 to 2.99% with threshed panicle compensation. The results also proved that the system measurement was approximately 20 times more efficient than manual measurement and that cloud computing dramatically reduced the system cost and improved the system flexibility. In conclusion, this study provides a novel and powerful tool for phenotyping yield-related traits that will benefit rice breeding and genetic research in the future.

## Data Availability Statement

The raw data supporting the conclusions of this article will be made available by the authors, without undue reservation.

## Author Contributions

CH and WL designed the research, performed the experiments, analyzed the data, and wrote the manuscript. ZZ, XH, JYe, JYa, LD, and XL contributed to the experiments. WY supervised the project and wrote the manuscript. All authors contributed to the article and approved the submitted version.

## Conflict of Interest

The authors declare that the research was conducted in the absence of any commercial or financial relationships that could be construed as a potential conflict of interest.

## Publisher’s Note

All claims expressed in this article are solely those of the authors and do not necessarily represent those of their affiliated organizations, or those of the publisher, the editors and the reviewers. Any product that may be evaluated in this article, or claim that may be made by its manufacturer, is not guaranteed or endorsed by the publisher.

## Supplementary Material

Supplementary Video 1 The system workflow and operation procedures of YTS-2.0.
